# A Comprehensive Review on Function of miR-15b-5p in Malignant and Non-Malignant Disorders

**DOI:** 10.3389/fonc.2022.870996

**Published:** 2022-05-02

**Authors:** Soudeh Ghafouri-Fard, Tayyebeh Khoshbakht, Bashdar Mahmud Hussen, Hazha Hadayat Jamal, Mohammad Taheri, Mohammadreza Hajiesmaeili

**Affiliations:** ^1^ Department of Medical Genetics, School of Medicine, Shahid Beheshti University of Medical Sciences, Tehran, Iran; ^2^ Men’s Health and Reproductive Health Research Center, Shahid Beheshti University of Medical Sciences, Tehran, Iran; ^3^ Department of Pharmacognosy, College of Pharmacy, Hawler Medical University, Erbil, Iraq; ^4^ Center of Research and Strategic Studies, Lebanese French University, Erbil, Iraq; ^5^ Department of Biology, College of Education, Salahaddin University, Erbil, Iraq; ^6^ Urology and Nephrology Research Center, Shahid Beheshti University of Medical Sciences, Tehran, Iran; ^7^ Institute of Human Genetics, Jena University Hospital, Jena, Germany; ^8^ Skull Base Research Center, Loghman Hakim Hospital, Shahid Beheshti University of Medical Sciences, Tehran, Iran; ^9^ Critical Care Fellowship, Department of Anesthesiology, Loghman Hakim Hospital, Shahid Beheshti University of Medical Sciences, Tehran, Iran

**Keywords:** miR-15b-5p, cancer, biomarker, expression, malignance

## Abstract

miR-15b-5p is encoded by *MIR15B* gene. This gene is located on cytogenetic band 3q25.33. This miRNA participates in the pathogenesis of several cancers as well as non-malignant conditions, such as abdominal aortic aneurysm, Alzheimer’s and Parkinson’s diseases, cerebral ischemia reperfusion injury, coronary artery disease, dexamethasone induced steatosis, diabetic complications and doxorubicin-induced cardiotoxicity. In malignant conditions, both oncogenic and tumor suppressor impacts have been described for miR-15b-5p. Dysregulation of miR-15b-5p in clinical samples has been associated with poor outcome in different kinds of cancers. In this review, we discuss the role of miR-15b-5p in malignant and non-malignant conditions.

## Introduction

microRNAs (miRNAs) are a category of non-coding RNA with sizes about 20-24 nucleotide which participate in post-transcriptional control of gene expression (1). This effect is exerted through modulation of stability and translation of mRNAs. The primary transcripts produced by RNA polymerase II have 5’-cap and 3’-polyadenylated tail. Then, Drosha ribonuclease III enzyme cleaves this transcript to make the stem-loop precursor miRNA with an estimated size of 70 nucleotides (2). Finally, this transcript is processed by the Dicer ribonuclease to make the mature miRNA which can be combined into the RNA-induced silencing complex. Through incorporation into this complex, miRNAs can recognize their target transcript in a base pairing-dependent process resulting in suppression of translation or destabilization of transcript (3).

*MIR15B* gene is located on cytogenetic band 3q25.33 and encodes hsa-mir-15b. This miRNA participates in the pathogenesis of several cancers as well as non-malignant conditions, including cardiovascular disorders, neuropsychiatric diseases and metabolic conditions. This miRNA has been reported to exert oncogenic or tumor suppressor effects in different malignancies. We have searched the literature and discussed the role of miR-15b-5p in malignant and non-malignant conditions.

## miR-15b-5p in Cancers

### Cell Line Studies

In bladder cancer cell lines, the long non-coding RNA (lncRNA) MAGI2-AS3 acts as a molecular sponge for miR-15b-5p. In fact, MAGI2-AS3 exerts its tumor suppressor role in bladder cancer through decreasing level of this miRNA. Meanwhile, miR-15b-5p has been found to target the tumor suppressor gene CCDC19. Taken together, MAGI2-AS3/miR-15b-5p/CCDC19 axis has been revealed to regulate progression of bladder cancer (4).

An *in vitro* experiment in breast cancer cells has shown that miR-15b-5p silencing could restrain cell proliferation and invasiveness and induce apoptosis, while its up-regulation has exerted the opposite impacts. Notably, heparanase-2 (HPSE2) has been acknowledged as the target of miR-15b-5p in breast cancer cells, through which this miRNA applies its effect (5).

In cervical cancer cells, level of the tumor suppressor lncRNA FENDRR has been shown to be decreased. This lncRNA has binding sites for miR-15a-5p and miR-15b-5p, two miRNAs that can down-regulate expression of Tubulin alpha1A (TUBA1A). Taken together, FENDRR/miR-15a/b-5p/TUBA1A molecular route has been proved to regulate progression of cervical cancer (6).

Expression of miR-15b-5p has been reported to be surged in colon cancer cells. Treatment of HT-29 cells with a PNA against miR-15b-5p has been shown to reduce cell proliferation and activate the pro-apoptotic pathway (7). Another research in colon cancer cells has displayed that SIRT1 suppresses metastatic ability of cells through decreasing expression of miR-15b-5p. In fact, SIRT1 disrupts the regulatory effect of AP-1 on activation of expression of miR-15b-5p *via* deacetylating this activation factor. miR-15b-5p can target the transcript of a central enzyme in the fatty acid oxidation, namely acyl-CoA oxidase 1 (ACOX1). Taken together, SIRT1/miR-15b-5p/ACOX1 axis has been identified as a functional route in regulation of metastatic ability of colorectal cancer cells (8).

**Figure 1:**
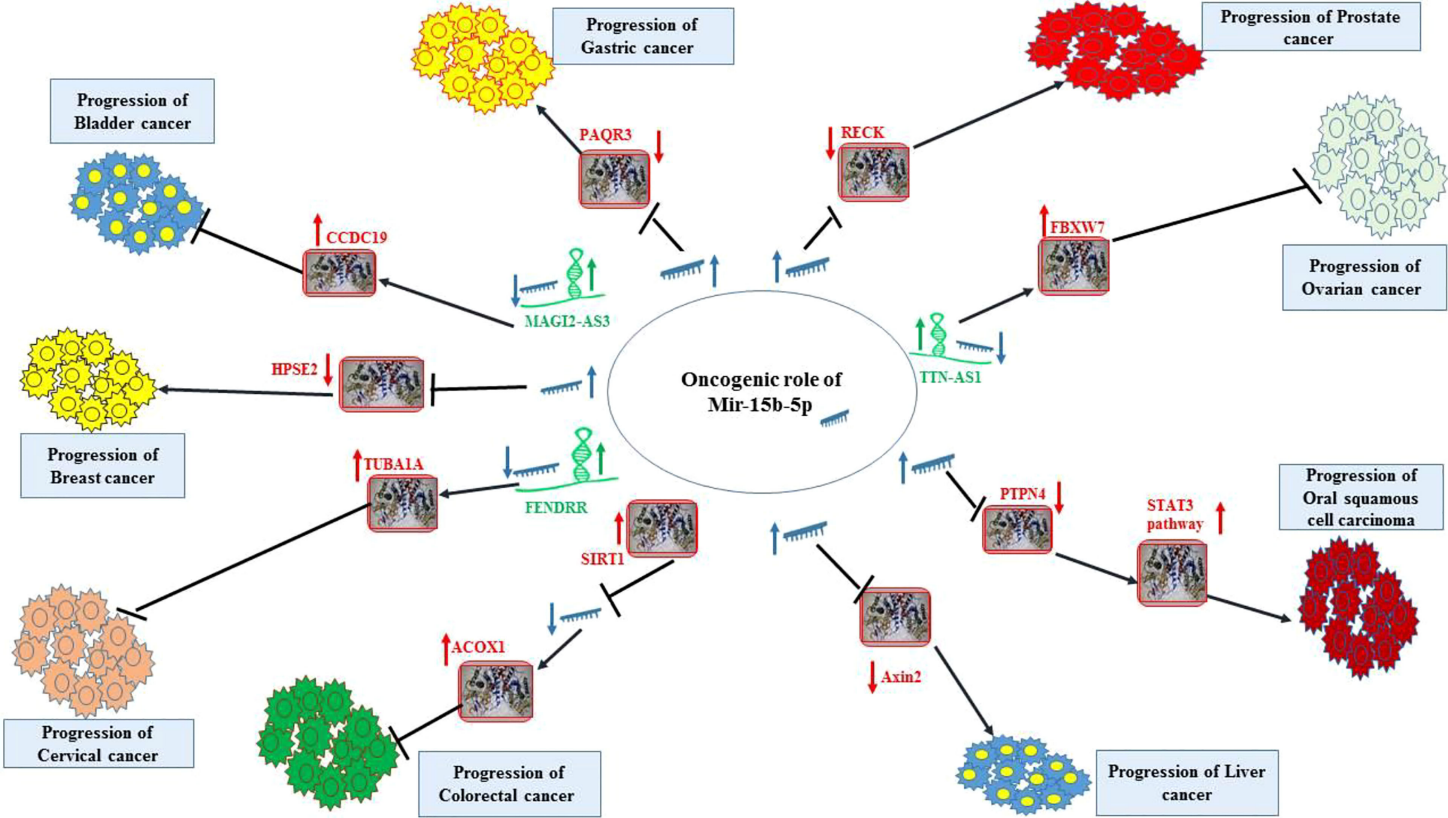
Oncogenic effect of miR-15b-5p in bladder, breast, cervical, colorectal, liver, oral, ovarian, prostate and gastric cancers. Detailed information about the conducted experiments is shown in **Table 1**.

**Figure 1** displays the oncogenic role of miR-15b-5p in bladder, breast, cervical, colorectal, liver, oral, ovarian, prostate and gastric cancers.

**Table 1:** Summary of cell line studies on the role of miR-15b-5p in cancers (Δ, knock-down or deletion; MET, mesenchymal-epithelial transition).

Tumors	Interactions	Cell line	Function	Reference
Bladder cancer	MAGI2-AS3 and CCDC19	EJ, T24 and RT4, SV-HUC-1	↑↑ MAGI2-AS3 (which sponges mir-15b-5p): ↓ Proliferation, ↓ migration and ↓ invasion	(4)
Breast cancer	HPSE2	MDA-MB-231, MCF-7, 293T	Δ miR-15b-5p: ↓ proliferation, ↓ colony formation, ↓ migration and ↓ invasion, ↑ apoptosis	(5)
Cervical cancer	FENDRR, TUBA1A	HeLa, SiHa, CaSki, C33A, Ect1-E6E7	↑↑ FENDRR (which sponges mir-15b-5p): ↓ proliferation, ↓ migration and ↓ invasion, and ↓ cell viability, and ↑ apoptosis	(6)
			↑↑ mir-15b-5p: ↑ proliferation, ↑ migration and ↑ invasion, and ↑ cell viability, and ↓ apoptosis	
Colorectal cancer	NF-κB1 and IKK-α	NCM460, SW620, HCT116, DLD1, SW1116	↑↑ miR-15b-5p: ↑ sensitivity to 5-FU and ↑ apoptosis	(9)
	_	HT-29 cell line	R8-PNA-a15b molecule treatment: ↓ miR-15b-5p levels and ↑ inhibition of HT-29 cell growth associated with pro-apoptotic effects	(7)
	SIRT1, AP-1, ACOX1	HCT116, SW480, SW620, LoVo, Caco-2, HT-29	↑↑ SIRT1: ↓ migration and invasion and suppresses mir-15b-5p transcription *via* AP-1	(8)
	IL-17A, PD-L1, P65, NRF1	CT26, MC38, SW1116, HT29, SW480, SW620	↑↑ miR-15b-5p: ↓ PD-L1 protein level and ↑ anti-PD-1 sensitivity	(10)
	CERS6-AS1	FHC, Caco-2, T84, HCT-15	Δ CERS6-AS1 (whish sponges miR-15b-5p): ↓ proliferation, ↓ migration, ↓ invasion, ↓ EMT, and ↓ stemness	(11)
Gastric cancer	PAQR3	AGS, BGC-823, SGC-7901, MGC-803	↑↑ miR-15b-5p: ↑ migration and ↑ invasion	(12)
Glioblastoma multiforme	_	U251	Combo-therapy using PNA-a15b and SFN *via* interfering with miR-15b-5p could be used as a treatment for Glioblastoma multiforme to stimulate apoptosis.	(13)
Hepatocellular carcinoma	OIP5, AKT/mTORC1 and β-catenin signaling pathways	HepG2, Hep3B, SK-HEP-1, Chang liver and THLE2, Huh7	Δ OIP5 (a target of mir-15b-5p): ↓ migration, ↓ invasion and ↓ EMT process *via* mTORC1 and GSK-3β/β-catenin signaling	(12)
	H19 and CDC42/PAK1 signaling pathway	HepG2, SMMC-7721, Bel-7402, Huh-7, WRL-68, 293T	Δ H19 (which sponges mir-15b-5p): ↓ proliferation, migration, invasion, EMT and CDC42/PAK1 signaling pathway and ↑ apoptosis	(14)
	Rab1A	SMMC-7721, HepG2, Hep3B, HL-7702	↑↑ miR-15b-5p: ↓ cell growth, ↑ endoplasmic reticulum stress and apoptosis	(15)
			Δ miR-15b-5p: ↑ proliferation and ↓ apoptosis	
Laryngeal cancer	TXNIP	HEP-2	↑↑ miR-15b-5p: ↑ cell growth *via* targeting TXNIP	(16)
Liver cancer	Axin2	HepG2 and Huh7, Hep3B and HCCLM3	↑↑ miR-15b-5p: ↑	(14)
			Proliferation and ↑ invasion	
Neuroblastoma	MYCN	SK‐N‐BE (2), NB‐19, SH‐EP Tet21N, CHLA‐136	↑↑ miR-15b-5p: ↓ proliferation, ↓ migration, and ↓ invasion of NB cells	(17)
	SNHG16, PRPS1	neuroblastoma cells	Δ SNHG16 (which sponges mir-15b-5p): ↓ proliferation, and ↑ G0/G1 phase arrest	(18)
Non-small cell lung cancer	MEG8 and PSAT1	16HBE, A549, H1299, H1975, SPC-A1, and PC-9	Δ MEG8 (which sponges mir-15b-5p): ↓ proliferation, ↓ migration, and ↓ invasion	(19)
Oral squamous cell carcinoma	PTPN4, STAT3 pathway	SCC-4, UM-1, CAL-27, OSC-4	Δ mir-15b-5p: ↓ proliferation, ↓ migration, and ↓ invasion and ↑ apoptosis	(20)
Oral tongue squamous cell carcinoma	TRIM14	SCC25	↑↑ miR-15b: ↑ MET phenotypes and ↓ cisplatin-resistance *via* targeting TRIM14	(21)
Osteosarcoma	PDK4	hFOB1.19, MNNG-HOS, Saos-2, MG63, U-2OS	↑↑ miR-15b-5p: ↓ proliferation and the Warburg effect by suppressing PDK4 expression	(22)
	TRPM2-AS and PPM1D	OS cells	Δ TRPM2-AS (which sponges mir-15b-5p): ↓ viability, ↓ proliferation, ↓ migration and ↑ apoptosis	(23)
Ovarian cancer	TTN-AS1, FBXW7	A2780, OVCA429, IOSE80	↑↑ TTN-AS (which sponges mir-15b-5p): ↓ proliferation and ↓ colony formation, ↑ apoptosis	(24)
Prostate cancer	RECK	PCa cell lines (PC3 and 22RV1)	Δ miR-15b-5p: ↓ cell growth and invasion	(25)
	PVT1 and NOP2	DU 145, PC-3, RWPE-1	↑↑ PVT1 (which sponges mir-15b-5p): ↑ migration and ↑ invasion	(26)
Thyroid carcinoma	GDI2, MMP2 and MMP9	FTC133, SW1736, K1, Nthy-ori3-1	↑↑ mir-15b-5p: ↓ proliferation and ↓ invasion	(27)

↑ Up-regulation; ↓ Down-regulation.

In contrast to the previously mentioned experiment in colorectal cancer cells (7), Zhao et al. have shown that miR-15b-5p has a tumor suppressor impact in this cancer. Notably, miR-15b-5p can enhance 5-fluorouracil (5-FU)-induced apoptosis in these cells and reversed the resistance of colorectal cancer cells to this therapeutic agent. Mechanistically, miR-15b-5p exerts this impact through modulating activity of the NF-κB signaling *via* decreasing NF-κB1 and IKK-α levels. miR-15b-5p has been found to target the anti-apoptosis transcript XIAP (9).

**Figure 2:**
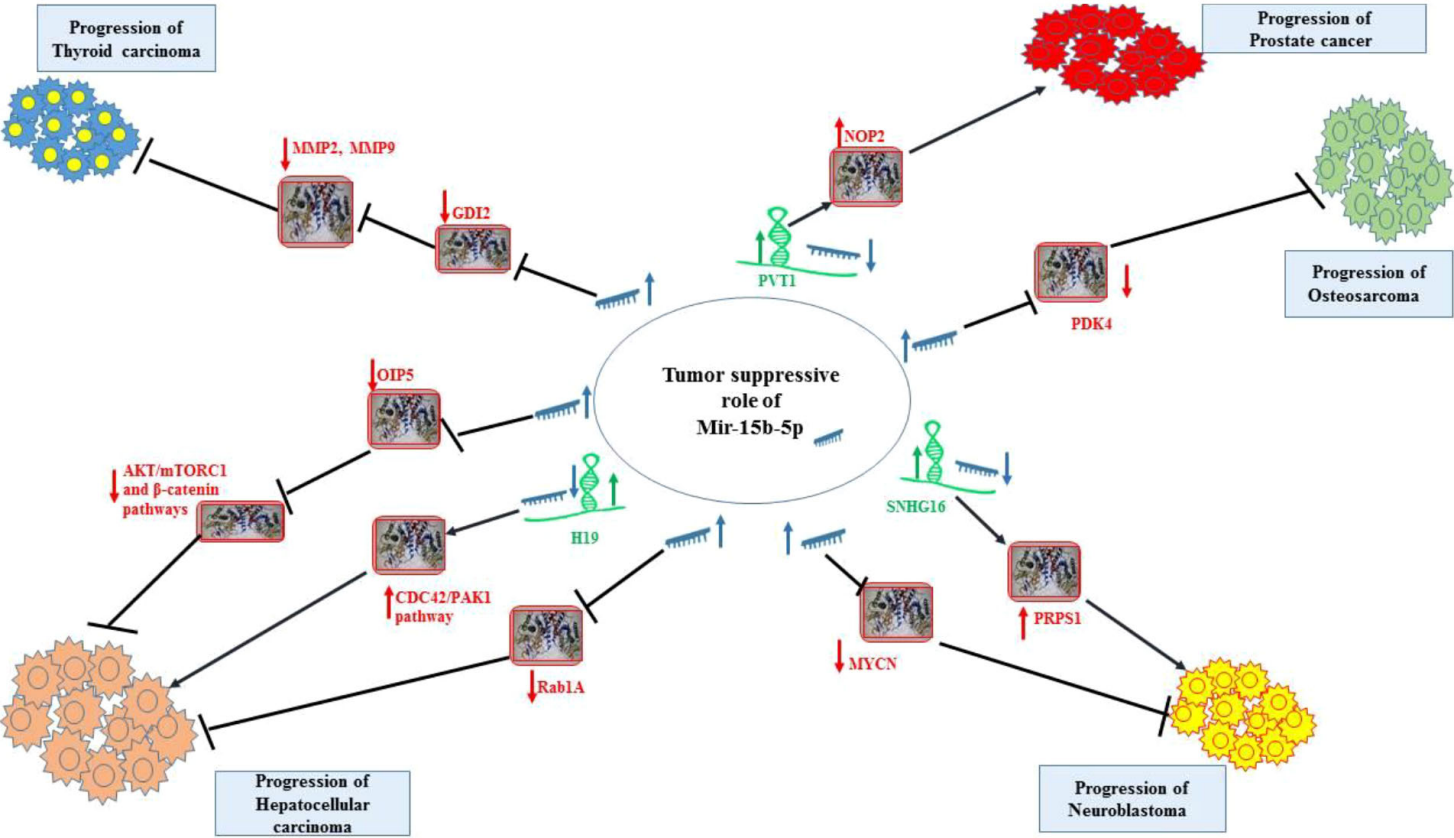
Tumor suppressor role of miR-15b-5p in thyroid cancer, hepatocellular carcinoma, neuroblastoma, osteosarcoma and prostate cancer. Detailed information about the conducted experiments is shown in **Table 1**.

*In vitro* experiments in neuroblastoma cells have shown that up-regulation of miR-15a-5p, miR-15b-5p or miR-16-5p can reduce expression of MYCN transcript and N-Myc protein. On the other hand, suppression of these miRNAs could lead to enhancement of MYCN transcripts and N-Myc protein level, along with increasing half-life of its mRNA. The interaction between these miRNAs and MYCN mRNA has been proved through conducting immunoprecipitation and luciferase reporter assays. Notably, up-regulation of these miRNAs has diminished proliferation, migration, and invasiveness of neuroblastoma cells (17). **Figure 2** shows tumor suppressor role of miR-15b-5p in thyroid cancer, hepatocellular carcinoma, neuroblastoma, osteosarcoma and prostate cancer.

### Animal Studies

Lovat et al. have produced miR-15b/16-2 knockout mice for the purpose of identification of the role of this cluster. This intervention has led to development of B-cell lymphomas by age 15–18 month possibly though modulation of expression of Cyclins D2 and D1, and IGF1R. These genes participate in the regulation of proliferation and antiapoptotic pathways. Taken together, this cluster has been shown to have a tumor suppressor role in mice models of B-cell lymphoma (28).

In xenograft models of bladder cancer, up-regulation of MAGI2-AS3 has reduced tumor volume possibly through decreasing expression of miR-15b-5p (4). Up-regulation of FENDRR, another miR-15b-5p-sponging lncRNA has exerted similar effects in xenograft models of cervical cancer (6). In colorectal cancer cells, a single study has shown that over-expression of miR-15b-5p improves sensitivity of cells to 5-FU (9). On the other hand, another study has indicated that SIRT1 decreases metastasis through suppression of miR-15b-5p transcription (8). Moreover, miR-15b-5p has been demonstrated to decrease expression of PD-L1, suppress tumorigenic potential of colorectal cancer cells and increase anti-PD-1 sensitivity in colitis-associated cancer and APC^min/+^ models of colorectal cancer (10).

In an animal model of osteosarcoma, over-expression of miR-15b-5p has been associated with reduced cell proliferation (22). **Table 2** shows summary of animal studies on the role of miR-15b-5p in cancers.

**Table 2:** Summary of animal studies on the role of miR-15b-5p in cancers (Δ, knock-down or deletion).

Tumors	Animals	Results	Reference
Bladder cancer	4-week-old female BALB/C nude mic	↑↑ MAGI2-AS3: ↓ tumor volume and↓ tumor weight	(4)
Breast cancer	5-week-old female BALB/C nude mice	Δ miR-15b-5p: ↓ tumorigenic ability	(5)
Cervical cancer	6-week-old male BALB/C nude mice	↑↑ FENDRR (which sponges mir-15b-5p): ↓ tumor volume and ↓ tumor weight	(6)
Colorectal cancer	Four-week-old female athymic nude mice	↑↑ miR-15b-5p: ↑ sensitivity of colon cancer cells to 5-FU and ↑ apoptosis *via* the NF-κB pathway	(9)
	4-6 weeks old BALB/c nude mice	↑↑ SIRT1: ↓ metastasis by suppressing mir-15b-5p transcription *via* AP-1	(8)
	female BALB/c mice with two different groups control and blocking miR-15b-5p groups	Δ miR-15b-5p: ↑ tumorigenesis and ↑ PD-L1 levels	(10)
	BALB/c nude mice	Δ CERS6-AS1 (whish sponges miR-15b-5p): ↓ tumor growth	(11)
Hepatocellular carcinoma	Four-week-old female BALB/c nude mice	Δ OIP5 (a target of mir-15b-5p): ↓ tumor growth and ↓ metastasis	(12)
	Four-week-old male BALB/C nude mice	↑↑ miR-15b-5p: ↓ tumor growth, ↓ tumor volume and ↓ tumor weight	(15)
Neuroblastoma	Six‐week‐old NOD mice	↑↑ miR-15b-5p: ↓ tumor size and ↓ tumor weight ​	(17)
Non-small cell lung cancer	Balb/c nude mice	Δ MEG8 (which sponges mir-15b-5p): ↓ tumor growth	(19)
Oral squamous cell carcinoma	5-week-old female specific-pathogen-free mice	Δ mir-15b-5p: ↓ tumor growth and ↓ metastasis	(20)
Osteosarcoma	5-week-old male BALB/C nude mice	↑↑ miR-15b-5p: ↓ proliferation	(22)
Prostate cancer	PC3 xenograft tumor model	Δ miR-15b-5p: ↓ tumor volume and ↓ tumor weight	(25)

↑ Up-regulation; ↓ Down-regulation.

### Human Studies

Expression assays in clinical samples obtained from patients with bladder cancer, breast cancer, gastric cancer, oral squamous cell carcinoma and prostate cancer have shown up-regulation of miR-15b-5p. On the other hand, this miRNA has been found to be down-regulated in head and neck cancer squamous cell carcinomas, neublastoma and thyroid cancer samples. Different studies in colorectal cancer and hepatocellular carcinoma sample have shown contradictory expression patterns (**Table 3**). Moreover, dysregulation of expression of miR-15b-5p has been associated with poor clinical outcome in bladder cancer, breast cancer, head and neck/oral squamous cell carcinoma, hepatocellular carcinoma and neuroblastoma.

**Table 3:** Summary of human studies on the role of miR-15b-5p in cancers (NB, Neuroblastoma; OS, Overall survival; ANCTs, adjacent non-cancerous tissues; TNM, tumor‐node‐metastasis; MSS, microsatellite stable; CRC, colorectal cancer; RFS, relapse-free survival; HCC, Hepatocellular carcinoma).

Tumors	Specimens	Expression (Tumor vs. Normal)	Kaplan-Meier analysis (as a result of dysregulation in mir-15b-5p)	Multivariate/Univariate cox regression	Clinicopathologic characteristics	Method by which RNA was detected	Reference
Bladder cancer	10 patients with and without BC included 3 healthy persons and 7 patients with other urologic diseases	upregulated	_	_	_	ExiLENT SYBR^®^ Green master mix	(29)
	TCGA database 58 pairs of tumor tissues and ANCTs	upregulated	Poorer OS	_	_	PrimeScript RT-PCR kit	(4)
Breast cancer	6 pairs of tumor tissues and ANCTs TCGA databases	upregulated	Poorer OS	_	_	_	(5)
Cervical cancer	53 pairs of tumor tissues and ANCTs	Downregulation of FENDRR (which sponges mir-15b-5p)	_	_	_	SYBR Green kit	(6)
Colorectal cancer	23 pairs of tumor tissues and ANCTs TCGA database	downregulated	_	_	_	TransStart SYBR Green supermix	(9)
Colorectal cancer	94 tumor tissues	downregulation in SIRT1 which suppresses mir-15b-5p transcription *via* AP-1	_	_	_	_	(8)
	110 pairs of tumor tissues and ANCTs TCGA database: MSS CRC samples	downregulated	_	_	_	_	(10)
	GEPIA database	upregulation of CERS6-AS1 (which sponges mir-15b-5p)	_	_	_	_	(11)
Gastric cancer	40 pairs of tumor tissues and ANCTs 100 patients and 100 healthy controls	upregulated	_	_	degree of tumor invasion and lymph node metastasis and distant metastasis	PrimeScript™ RT reagent kit	(12)
Head and neck cancer squamous cell carcinomas	43 HNSCC patient in explorative phase	downregulated	Shorter locoregional RFS	miR-15b-5p was found to be an independent predictive factor of LRC in HNSCC patients.	_	TaqMan stem-loop	(30)
	51 HNSCC patient in validation phase						
Hepatocellular carcinoma	TCGA and GEO databases	upregulated	_	_	_	_	(31)
	991 HCC and 456 adjacent non-HCC tissue samples						
	GEO database (GSE36411: 42 pairs of tumor tissues and ANCTs)	Upregulation of OIP5 (a target of miR-15b-5p)	_	_	_	_	(12)
	46 pairs of tumor tissues and ANCTs	downregulated	_	_	_	SYBR Green	(14)
	Phase I: 6 pairs of tumor tissues and ANCTs (from 6 HCC patients)	Overexpression in tumor tissues and preoperative plasmas, and downregulation in postoperative plasma	_	_	_	ALL-in-One™ miRNA qRT-PCR Detection Kit	(32)
	Phase II: 10 patients						
	Phase III: 37 HCC patients, 29 cirrhosis patients, and 31 healthy controls						
	28 pairs of tumor tissues and ANCTs	upregulated	_	_	_	SYBR Premix Ex Taq II on an FTC-3000TM System	(15)
Hepatocellular carcinoma (HBV-related type)	GEO database GSE27462 (5 pairs of tumor tissues and ANCTs) GSE76903 (20 pairs of tumor tissues and ANCTs) GSE121248 (70 pairs of tumor tissues and ANCTs)	upregulated	Poorer OS	_	_	_	(33)
Liver cancer	69 pairs of tumor tissues and ANCTs	upregulated	Poorer OS	_	TNM stage and tumor capsular infiltration	SYBR Premix Ex Taq	(14)
Neuroblastoma	Two cohort:	downregulated	Poorer OS	_	_	SYBR green mix (Bio-Rad) for mRNA expression or TaqMan Universal Fast PCR master mix	(17)
	88 NB patients and 105 NB patients						
	46 neuroblastoma samples and 28 normal tissues	downregulated	_	_	_	_	(18)
Non-small cell lung cancer	37 pairs of tumor tissues and ANCTs	downregulated	_	_	_	_	(19)
Oral squamous cell carcinoma	TCGA database	upregulated	Poorer OS	_	tumor stage, TNM stage, and tumor metastasis	SYBR Premix Ex Taq II	(20)
	37 pairs of tumor tissues and ANCTs						
Ovarian cancer	TCGA and genotype-tissue expression (GTEx) databases	downregulation in TTN-AS1 which sponges mir-15b-5p	_	_	_	_	(24)
Prostate cancer	TCGA database:	upregulated	_	_	age and Gleason score of patients with PCa	_	(25)
	495 patients and 52 pairs of tumor tissues and ANCTs						
Squamous cell carcinoma	10 patients and 30 healthy controls	downregulated	_	_	_	_	(34)
Thyroid carcinoma	Cancer Genome Atlas project database: 509 patients and 58 healthy controls	downregulated	Poorer OS	_	_	_	(27)

## Role of miR-15b-5p in Non-Malignant Conditions

### Cell Line Studies

*In vitro* experiments in vascular smooth muscle cells (VSMCs) have shown that up-regulation of miR-15b-5p suppresses cell proliferation and induces apoptosis, while its knock down leads to opposite results. These effects are possibly mediated through suppression of ACSS2. Transfection of these cells with miR-15b-5p mimic or inhibitor has led to down-regulation and up-regulation of ACSS2 and PTGS2, respectively. Taken together, miR-15b-5p may increase apoptosis of aortic VSMCs and suppress their proliferation through influencing ACSS2/PTGS2 axis, thus participating in the pathoetiology of abdominal aortic aneurysm (35).

miR-15b-5p has also been shown to mediate the anti-amyloid effect of curcumin in an *in vitro* model of Alzheimer’s disease through influencing expression of the amyloid precursor protein (36). Moreover, the antiangiogenic effect of isopimpinellin has been attributed to its impact on induction of miR-15b-5p expression and subsequent down-regulation of angiogenic stimulators (37).

In addition, miR-15b-5p has been shown to mediate the effects of LINC00473 in cerebral I/R injury. Experiments in a cellular model of cerebral I/R injury has shown down-regulation of LINC00473 in these cells. Up-regulation of this lncRNA has reversed the effects of oxygen glucose deprivation/reperfusion on cell viability and apoptosis as well as ROS levels. Mechanistically, LINC00473 acts as a molecular sponge for miR-15b-5p and miR-15a-5p and regulates expression of SRPK1 (38). **Table 4** shows summary of cell line studies on the role of miR-15b-5p in non-malignant conditions.

**Table 4:** Summary of cell line studies on the role of miR-15b-5p in non-malignant conditions (Δ, knock-down or deletion; DOX, doxorubicin; H2S, Hydrogen sulfide; HG, High glucose; SHF, secondary hair follicle; ER, endoplasmic reticulum; EVs, extracellular vesicles).

Disease type	Interactions	Cell line	Function	Reference
Abdominal aortic aneurysm	ACSS2 and PTGS2	Human aortic VSMCs (T/G HA-VSMC cell line)	↑↑ miR-15b-5p: ↓ proliferation and ↑ apoptosis of aortic VSMCs *via* targeting the ACSS2/PTGS2 axis	(35)
Alzheimer’s disease	amyloid precursor protein and amyloid-β	swAPP695-HEK293 cells and HEK293	Curcumin treatment: ↑ mir-15b-5p and ↓ amyloid precursor protein and ↓ amyloid-β	(36)
Angiogenesis	_	Human Umblical Vein Endothelial Cell (HUVEC)	Isopimpinellin: ↓ proliferation, ↓ invasion, ↓ migration, and tube formation *via* increasing mir-15b-5p levels and decreasing angiogenic stimulators	(37)
Asthma	YAP1	ASM cells	↑↑ miR-15b-5p: ↓ proliferation, migration, inflammatory response, and ECM deposition of TNF-α-induced ASM cells	(39)
Atherosclerosis	circCHFR and GADD45G	HUVECs	Upregulation of miR-15b-5p was found to reduce apoptosis, proinflammatory cytokine secretion, and improved cell survival *via* targeting GADD45G.	(40)
Cerebral I/R injury	LINC00473, SRPK1	Neuro-2a (N2a) cells	↑↑ LINC00473 (which sponges mir-15b-5p): ↑ cell viability, ↓ apoptosis and ↓ ROS level induced by OGD/R	(38)
Clopidogrel-induced liver injury	TLK1	HepG2 cells	Clopidogrel treatment: ↓ miR-15b and its target TLK1, which shows other molecules are involved in the regulation of TLK1 expression as a result of exposure to clopidogrel.	(41)
Coronary artery disease	AKT3	Human umbilical vein endothelial cells (HUVECs)	↑↑ miR-15b-5p: ↓ migration and ↓ proliferation of endothelial cells	(42)
			Δ miR-15b-5p: ↑ migration and ↑ proliferation of endothelial cells	
Coronary atherosclerotic heart disease	MALAT1 and MAPK1, mTOR signaling pathway	HEK 293T cells	Δ MALAT1 (which sponges mir-15b-5p): ↑ cell viability, ↑ autophagy and ↓ development of CAD	(43)
Dexamethasone induced steatosis	ENST00000608794, PDK4	dexamethasone treated HepG2 cell lines	Δ ENST00000608794 (which sponges miR-15b-5p): ↓ dexamethasone induced steatosis	(44)
			↑↑ miR-15b-5p: ↓ dexamethasone induced steatosis	
Diabetic foot ulcers	IKBKB and WEE1	human keratinocytes	S. aureus: ↑ miR-15b-5p levels	(45)
			↑↑ miR-15b-5p: ↓ DNA repair and ↓ inflammatory response	
Diabetic nephropathy	JNK and Akt/mTOR pathway	HK-2 and HKC-5 cells	High glucose treatment: ↓ expression of miR-15b-5p in HK-2 cells	(46)
			↑↑ miR-15b-5p: ↓ High glucose-induced apoptosis in HK-2 cells	
	BCL-2	Mouse MCs (CRL1927) and human embryonic kidney (HEK) 293 cells	High glucose treatment: ↑ miR-15b-5p expression in mouse MCs, so ↑ mouse MC apoptosis by targeting BCL-2	(47)
Diabetic nephropathy	CDKN2B-AS1 and WNT2B	HMCs	Δ miR-15b-5p: ↑ viability, ↑ cell cycle progression, ↑ ECM accumulation, ↑ inflammatory response	(48)
	PDK4 and VEGFA	MPC5 cells	High-glucose treatment: ↓ mir-15b-5p in podocytes	(49)
			↑↑ EVs-derived miR-15b-5p: ↓ MPC5 cell apoptosis and ↓ inflammation *via* reducing PDK4 and VEGFA	
Diabetic retinopathy	circ_001209, COL12A1	human retinal vascular endothelial cells (HRVECs)	High-glucose treatment: ↑ circ_001209 (which sponges miR-15b-5p) levels, thus ↑ COL12A1 (a target of miR-15b-5p) levels	(50)
			↑↑ miR-15b-5p: ↓ invasion, ↓ migration and ↓ tubular formation induced by HG	
Diabetic retinopathy	TNFα, SOCS3 and IGFBP-3 l	Human REC	miR-15b was found to have a role in the inhibition of insulin resistance by decreased TNFα and SOCS3 signaling and increased IGFBP-3 levels, resulting in REC protection from hyperglycemia-induced apoptosis.	(51)
DOX-induced cardiotoxicity	Bmpr1a	H9c2 cardiomyocytes	↑↑ miR-15b-5p: ↑ DOX-induced apoptosis, ↑ oxidative stress and ↑ mitochondria damage	(52)
Endoplasmic reticulum stress mediated neurons apoptosis	Rab1A	HT22 cells	Sevoflurane exposure: ↓ cell viability, and ↑ apoptosis and ↑ ER stress *via* increasing mir-15b-5p levels, thus inhibiting Rab1A	(53)
Fracture	HCAR, VEGF and MMP13	BMSCs	HCAR sponges miR-15b-5p to regulate VEGF and MMP13, so induces endochondral bone repair in hypertrophic chondrocyte.	(54)
High glucose-induced podocyte injury	Sema3A	mouse podocytes	↑↑ mir-15b-5p: ↓ apoptosis, ↓ oxidative stress, and ↓ inflammatory response	(55)
Inductive property of DPCs in cashmere goat	lncRNA-599547, Wnt10b	dermal papilla cells (DPCs) of passage 3 of cashmere goat SHF	lncRNA-599547 (which sponges miR-15b-5p) showed strongly high levels in dermal papilla of cashmere goat SHF.	(56)
Myocardial infarction	circ-Ttc3, Arl2	cardiomyocytes and cardiac fibroblasts	High levels of f circ-Ttc3 (which sponges miR-15b) was found to protect cardiomyocytes against ischemia-related apoptotic death.	(57)
Necroptosis and inflammation	TGFBR3, TGF-β pathway	HD11 and DT40	H2S exposure: ↑ oxidative stress and activates the TGF-β pathway by regulating miR-15b-5p/TGFBR3 axis miR-15b-5p is upregulated in H2S-induced necroptosis and inflammation.	(58)
Obstructive sleep apnea	PTGS1-NF-κB-SP1 signaling	human THP-1, HUVEC, and SH-SY5Y cell lines	Δ miR-15b-5p: ↑ IHR-induced oxidative stress and ↑ MAOA hyperactivity *via* targeting PTGS1-NF-κB-SP1 signaling in OSA patients	(59)
Osteoarthritis	LINC00662, GPR120	rat chondrocytes	LINC00662 is downregulated in osteoarthritis, so mir-15b-5p is upregulated and GPR120 is suppressed, thus inflammatory responses and apoptosis are induced.	(60)
Parkinson’s disease	LINC00943 and RAB3IP	SK-N-SH cells	Δ LINC00943 (which sponges miR-15b-5p): ↓ MPP+-caused decrease of cell viability so reduced MPP+-induced neuronal damage	(61)
	SNHG1 and GSK3β	1-methyl-4-phenylpyridinium ion (MPP+)-treated SH-SY5Y cells	↑↑ SNHG1 (which sponges miR-15b-5p): ↑ MPP+ -induced cellular toxicity, ↓ cell viability *via* miR-15b-5p/GSK3β axis	(62)
	Akt3	293T cells and the human dopaminergic neuroblastoma SH-SY5Y cells	↑↑ miR-15b-5p: ↑ apoptosis by targeting Akt3 in an MPP+-induced PD cell model	(63)
	SNHG1, SIAH1	SH-SY5Y	↑↑ miR-15b-5p: ↓ α-synuclein aggregation and ↓ apoptosis *via* targeting SIAH1	(64)
Severe acute respiratory syndrome coronavirus 2	viral RdRp	_	↑↑ miR-15b-5p: ↓ viral infection and ↓ proliferation by targeting the RNA template component of SARS-CoV-2 RdRp	(65)
Skeletal muscle atrophy	lncIRS1 and IRS1	DF‐1 cells	LncIRS1 (which sponges mir-15b-5p) was found to regulate myoblast proliferation and differentiation *in vitro via* increasing IRS1.	(66)
Tendon injury	circRNA-Ep400, FGF-1/7/9	293 T cells, fibroblasts and tenocytes	↑↑ M2 macrophage-derived circRNA-Ep400 (which sponges mir-15b-5p): ↑ fibrosis, ↑ proliferation, and ↑ migration	(67)

↑ Up-regulation; ↓ Down-regulation.

### Animal Studies

Animal studies have highlighted the role of miR-15b-5p in different cellular processes and disorders such as angiogenesis, coronary artery disease, diabetic nephropathy, diabetic retinopathy, myocardial I/R injury, necroptosis and inflammation, Parkinson’s disease and trachea inflammatory injury (**Table 5**). For instance, overexpression of miR-15b-5p has considerably suppressed arteriogenesis and angiogenesis in animal models through targeting AKT3. Remarkably, siRNA-mediated silencing of AKT3 has inhibited arteriogenesis and the rescue of blood perfusion following femoral ligation in animals (42). Another animal study has shown that silencing of the miR-15b-5p-sponging lncRNA MALAT1 decreases atherosclerotic process (43). miR-15b-5p has also been shown to affect diabetic nephropathy and retinopathy in animals. Assessment of transcriptome of high glucose-exposed mouse mesangial cells has shown the effect of miR-15b-5p and its downstream target BCL-2 in regulation of high glocose-induced apoptosis. Besides, db/db mice has been shown to have higher levels of urinary miR-15b-5p (47).

**Table 5:** Summary of studies on the role of miR-15b-5p in non-malignant conditions (Δ, knock-down or deletion; MDA, malondialdehyde; ECs, endothelial cells; ACR, Albumin-to-Creatinine Ratio; H2S, Hydrogen sulfide).

Disease Type	Animal models	Results	Reference
Angiogenesis	zebrafish embryos	Isopimpinellin: ↓ intersegmental vessels	(37)
Coronary artery disease	8-10-week-old male C57BL/6 mice Mice were received agomiR-15b, agomiR-NC, or cholesterol-conjugated AKT3 siRNA by multi-point injections.	miR-15b-5p expression was decreased, because of a reduced expression in EC layer of collaterals and miR-15b-5p was mainly derived from ECs.	(42)
		↑↑ miR-15b-5p: ↓ arteriogenesis and ↓ angiogenesis	
Coronary atherosclerotic heart disease	Six-week old male ApoE−/−mice	Δ MALAT1 (which sponges mir-15b-5p): ↓ atherosclerosis	(43)
Diabetic nephropathy	5 db/m mice and 5 db/db mice	Higher urine miR-15b-5p levels were found in db/db mice.	(47)
		Urinary EV miR-15b-5p levels were positively associated with urinary ACR.	
Diabetic retinopathy	80 Sprague–Dawley male rats	With increased levels of circ_001209 (which sponges miR-15b-5p) retinal thickness was thinner in diabetic rats, and apoptosis was enhanced.	(68)
Myocardial ischemia reperfusion injury	6-8 week-old male C57/B6 mice	Δ mir-15b-5p: ↓ arrhythmia, infarct extent and apoptosis, ↓ MDA content in the myocardial tissue by increasing levels of KCNJ2 (a target of mir-15b-5p)	(69)
Necroptosis and inflammation	40 one-day-old Ross 308 male broilers	H2S exposure: ↑ necroptosis and inflammation	(58)
Parkinson’s disease	five-week-old male C57BL/6 mice	Δ miR-15b-5p: ↓ MPTP-induced apoptosis by regulating Akt3	(63)
Skeletal muscle atrophy	1‐day‐old chicks	LncIRS1 (which sponges mir-15b-5p) was found to regulate muscle mass and muscle fibre cross‐sectional area.	(66)
Trachea inflammatory injury	Eighty one-day-old Ross 308 broilers divided into two groups (control group and H2S group)	H2S exposure: ↑ mir-15b-5p miR-15b-5p reduced ATF2 levels to mediate METs release, which induces trachea inflammatory damage	(70)

↑ Up-regulation; ↓ Down-regulation.

### Human Studies

Different experiments in human samples obtained from patients with acute mountain sickness, asthma-COPD overlap, coronary artery disease, diabetic foot ulcers, diabetic nephropathy, late pulmonary complications, obstructive sleep apnea and Parkinson’s disease have shown dysregulation of miR-15b-5p levels (**Table 6**).

**Table 6:** Summary of human studies on the role of miR-15b-5p in non-malignant conditions (CAD, coronary atherosclerotic heart disease; CCC, coronary collateral circulation; ACR, albumin-to-creatinine ratio; eGFR, Estimated Glomerular Filtration Rate; AMS, Acute mountain sickness; COPD, chronic obstructive pulmonary disease; ACO, asthma-COPD overlap; DN, diabetic nephropathy; OSA, obstructive sleep apnea; CPAP, continuous positive airway pressure; DFU, Diabetic foot ulcers; FS, foot skin).

Disease type	Numbers of clinical samples	Expression (Tumor vs. Normal)	Clinicopathologic characteristics of patients	Method by which RNA was detected	Reference
Acute mountain sickness	124 healthy men (75 AMS+ group and 49 AMS– group)	upregulated in AMS- group	_	iQ™5 Real-Time PCR Detection System	(71)
Alzheimer’s disease	50 AD patients and 50 healthy controls	no significant differences	_	_	(72)
Asthma-COPD overlap	Cohort 1: 6 patients with ACO and 6 patients with asthma	downregulated in ACO patients	_	miScript SYBR Green PCR Ki	(73)
	Cohort 2; 30 patients with asthma, 30 patients with COPD, or 30 patients with ACO				
Atherosclerosis	30 patients with atherosclerosis and 30 healthy controls	downregulated	_	SYBR Green PCR kit	(40)
Coronary artery disease	5 patients with poor CCC and 5 patients with good CCC	upregulated in patients with poor CCC	miR-15b-5p was associated with insufficient coronary collateral artery function.	SYBR Premix Ex Taq qRT-PCR assays	(42)
	20 patients with poor CCC and 18 patients with good CCC and 18 healthy controls				
Coronary atherosclerotic heart disease	GEO database (GSE18608: 10 CAD patients and 4 healthy controls	downregulated	_	SYBR green	(43)
	5 CAD patients and 5 healthy controls				
Diabetic foot ulcers	12 DFU and 12 FS specimens	upregulated in DFU	_	PerfeCTa^®^ SYBR^®^ Green SuperMix	(45)
	6 DFU and 6 FS specimens				
	(GEO database GSE80178)				
Diabetic nephropathy	85 type 2 diabetic patients and 39 healthy controls	upregulated	Urinary EV miR-15b-5p levels were found to be positively associated with urinary ACR, negatively associated with eGFR, and correlated with rapid decline in kidney function in humans.	_	(47)
	34 DN patients and 34 healthy controls	downregulated	_	SYBR Green	(48)
Late pulmonary complications	20 Sulfur mustard-exposed individuals and 20 healthy controls	no differences	_	_	(74)
Obstructive sleep apnea	Discovery cohort: 16 OSA Patients and 8 healthy controls	downredulated in OSA patients	miR-15b-5p was negatively associated with an apnea hypopnea index	NGS (Illumina MiSeq platform) and SYBR Green PCR kit	(59)
	Validation cohort: 20 Primary Snoring, 45 Treatment-Naïve				
	OSA Patients, and 13 OSA Patients on CPAP				
Parkinson’s disease	10 patients and 5 healthy controls	upregulated	_	ABI PRISM^®^ 7500 Sequence Detection System	(63)

This miRNA might participate in the pathoetiology of acute mountain sickness. Levels of miR-15b-5p in the saliva have been found to be higher in individuals being resistant to this condition compared to susceptible ones. Combination of levels of miR-134-3p and miR-15b-5p could discriminate between these two groups. Thus, salivary levels of miR-134-3p and miR-15b-5p have been suggested as non-invasive markers for prediction of acute mountain sickness prior to exposure to high altitude (71).

Although *in vitro* studies indicated possible role of miR-15b-5p in the pathogenesis of Alzheimer’s disease (36), serum levels of miR-15b-5p were not significantly different between patients with Alzheimer’s disease and healthy subjects (72).

miR-15b-5p has been among miRNA having lower expression in asthma-COPD overlap patients. This miRNA can distinguish between asthma-COPD overlap patients and individuals with either asthma or COPD. In fact, miR-15b-5p has been shown to be superior to other miRNAs in separation of patients with asthma-COPD overlap from similar conditions (73).

In some conditions, dysregulation of this miRNA has been associated with clinicopathological parameters. For instance, in patients with coronary artery disease, dysregulation of miR-15b-5p has been associated with insufficient coronary collateral artery function (42). Moreover, in diabetic nephropathy, Urinary exosomal levels of miR-15b-5p have been positively associated with urinary albumin-to-creatinine ratio, negatively associated with eGFR, and correlated with speedy failure in kidney function (47).

## Discussion

miR-15b-5p is an example of miRNAs with dual roels in the carcinogenesis. While it is a putative oncogenic miRNA in bladder cancer, breast cancer, gastric cancer, oral squamous cell carcinoma and prostate cancer, it has been found to be down-regulated in head and neck cancer squamous cell carcinomas, neublastoma and thyroid cancer samples as compared with corresponding non-cancerous samples (75). Moreover, in colorectal cancer and hepatocellular carcinoma, different studies have reported contradictory results.

This miRNA also participates in the pathogenesis of several non-malignant conditions, such as abdominal aortic aneurysm, Alzheimer’s disease, Parkinson’s disease, cerebral I/R injury, coronary artery disease, dexamethasone induced steatosis, diabetic complications and doxorubicin-induced cardiotoxicity.

miR-15b-5p has been shown to be sponged by several lncRNAs, namely MAGI2-AS3, H19, SNHG1, SNHG16, TTN‐AS1, PVT1, FENDRR, SSTR5−AS1, MALAT1, ENST00000608794, CDKN2B-AS1, LINC00473, LINC00662, LINC00943, LncRNA-599547 and CDKN2B-AS1 as well as the circular RNA Circ_001209. Thus, lncRNAs and circRNAs can affect expression of this miRNA. Other possible regulatory mechanisms for modulation of expression levels of miR-15b-5p should be clarified in future studies.

NF-κB, STAT3, AKT/mTORC1, CDC42/PAK1 and β-catenin signaling pathways are signaling pathways that mediate the effects of miR-15b-5p in the carcinogenesis. Notably, this miRNA could regulate response of cancer cells to 5-FU and anti-PD-1 drugs. Thus, therapeutics modalities affecting expression of miR-15b-5p can be considered as possible ways to combat resistance to anti-cancer agents. Evidence from *in vitro* and *in vivo* studies indicates that therapeutic intervention with miR-15-5p levels can significantly influence pathological processes. Moreover, disease-associated abnormal expression pattern of this miRNA in the affected tissues potentiates it as a diagnostic biomarkers. Particularly, in bladder cancer, breast cancer, head and neck cancers, liver cancer, neuroblastoma, oral squamous cell carcinoma and thyroid cancer, abnormal expression of miR-15-5p has been associated with poor clinical outcomes indicating the role of this miRNA as a prognostic biomarker. It is expected that therapeutic modalities affect expression of miR-15-5p and amend disease-associated dysregulation of this miRNA. Therefore, expression pattern of miR-15-5p can be used to monitor disease status and response to therapeutic options.

Since both oncogenic and tumor suppressor roles have been reported for miR-15-5p, different miR-15-5p-targeting therapeutic targets can been applied in the field of cancer therapy. In tissues that this miRNA exerts tumor suppressor roles, exogenous miR-15-5p can be used to inhibit cell proliferation or induce apoptosis. This goal can be achieved by administration of chemically synthesized miR-15-5p mimics to induce expression of endogenous mature double-stranded miR-15-5p to restore function of this miRNA. Viral vectors expressing miR-15-5p are appropriate vectors for delivery of this miRNA to tumor cells. On the other hand, when miR-15-5p exerts oncogenic roles, antisense oligonucleotides and miR-15-5p sponges can be used for suppression of level of this miRNA. Although these strategies are putative therapeutic modalities for treatment of cancer, they have not been applied in the clinical setting yet.

## Conclusion

While the prognostic impact of dysregulation of miR-15b-5p has been confirmed in different types of cancer, there is no explicit evidence for application of this miRNA as a diagnostic marker in cancers. Since miRNAs dysregulation in the circulation provides a potential way for early non-invasive diagnosis of cancer, future studies should focus on evaluation of expression levels of miR-15b-5p in different biofluids during the course of cancer to provide insights into diagnostic role of this miRNA in cancer.

## Author Contributions

SG-F wrote the manuscript and revised it. MT supervised and designed the study. TK, HJ, MH and BH collected the data and designed the figures and tables. All authors read and approved the submitted version.

## Funding

This study was financially supported by Grant from Medical School of Shahid Beheshti University of Medical Sciences.

## Conflict of Interest

The authors declare that the research was conducted in the absence of any commercial or financial relationships that could be construed as a potential conflict of interest.

## Publisher’s Note

All claims expressed in this article are solely those of the authors and do not necessarily represent those of their affiliated organizations, or those of the publisher, the editors and the reviewers. Any product that may be evaluated in this article, or claim that may be made by its manufacturer, is not guaranteed or endorsed by the publisher.
